# A functional MRI investigation of crossmodal interference in an audiovisual Stroop task

**DOI:** 10.1371/journal.pone.0210736

**Published:** 2019-01-15

**Authors:** Megan C. Fitzhugh, Peter S. Whitehead, Lisa Johnson, Julia M. Cai, Leslie C. Baxter, Corianne Rogalsky

**Affiliations:** 1 Interdisciplinary Neuroscience Graduate Degree Program, Arizona State University, Tempe, Arizona, United States of America; 2 Department of Speech and Hearing Science, Arizona State University, Tempe, Arizona, United States of America; 3 Keller Center for Imaging Innovation, Barrow Neurological Institute & St. Joseph’s Hospital and Medical Center, Phoenix, Arizona, United States of America; University of Montreal, CANADA

## Abstract

The visual color-word Stroop task is widely used in clinical and research settings as a measure of cognitive control. Numerous neuroimaging studies have used color-word Stroop tasks to investigate the neural resources supporting cognitive control, but to our knowledge all have used unimodal (typically visual) Stroop paradigms. Thus, it is possible that this classic measure of cognitive control is not capturing the resources involved in multisensory cognitive control. The audiovisual integration and crossmodal correspondence literatures identify regions sensitive to congruency of auditory and visual stimuli, but it is unclear how these regions relate to the unimodal cognitive control literature. In this study we aimed to identify brain regions engaged by crossmodal cognitive control during an audiovisual color-word Stroop task, and how they relate to previous unimodal Stroop and audiovisual integration findings. First, we replicated previous behavioral audiovisual Stroop findings in an fMRI-adapted audiovisual Stroop paradigm: incongruent visual information increased reaction time towards an auditory stimulus and congruent visual information decreased reaction time. Second, we investigated the brain regions supporting cognitive control during an audiovisual color-word Stroop task using fMRI. Similar to unimodal cognitive control tasks, a left superior parietal region exhibited an interference effect of visual information on the auditory stimulus. This superior parietal region was also identified using a standard audiovisual integration localizing procedure, indicating that audiovisual integration resources are sensitive to cognitive control demands. Facilitation of the auditory stimulus by congruent visual information was found in posterior superior temporal cortex, including in the posterior STS which has been found to support audiovisual integration. The dorsal anterior cingulate cortex, often implicated in unimodal Stroop tasks, was not modulated by the audiovisual Stroop task. Overall the findings indicate that an audiovisual color-word Stroop task engages overlapping resources with audiovisual integration and overlapping but distinct resources compared to unimodal Stroop tasks.

## Introduction

The human brain is capable of selectively attending to pertinent information, concurrently ignoring or inhibiting irrelevant information, overriding automatic responses, and correcting errors. Collectively, these specific abilities are referred to as cognitive control and have been the focus of dozens of research studies over the last several decades (for reviews see [1–4]).

A classic and widely-used index of cognitive control is the color-word Stroop task ([5] for a comprehensive review). In this task, participants are asked to identify the color of ink used for a written word, while ignoring the meaning of the word. In congruent trials, the meaning of a written word matches the font color in which it appears (e.g. “Red” presented in red ink), leading to reduced (i.e. facilitated) reaction times compared to identifying the font color of a neutral word (e.g. “Stage” presented in red ink). In incongruent Stroop trials, there is a mismatch between the meaning of the word and the font color (e.g. the word “red” presented in blue ink), resulting in increased reaction times and decreased accuracy (i.e. interference) compared to identifying the font color of a neutral word [5–11].

While the exact mechanisms and timing underlying these facilitation and interference findings continue to be debated [12–16], there is a general consensus that these well-replicated “Stroop effects” reflect increased demand for cognitive control resources during incongruent trials compared to the congruent trials [2,17,18]. The increase in cognitive control demands is due to activation of the semantic representation of the color-word that is read conflicting with the font color. Reading the word is a prepotent response even though the task is to name the font color; cognitive control is required to inhibit the influence of reading on the participant’s response. The degree to which the word’s meaning interferes (i.e. increased reaction time) is often used as a measure of cognitive control; less interference equates to better cognitive control. In this way, the color-word Stroop task has been used as proxy for cognitive control in a variety of patient populations [19–21] and across the lifespan, including school-aged children and older adults [22–25].

The neural resources supporting cognitive control in the visual color-word Stroop task are also well studied [14,18,26–33]. Systematic reviews and meta-analyses of neuroimaging studies identify a consistent network including bilateral anterior cingulate and supplementary motor areas, left inferior frontal regions, and bilateral parietal regions [34–38]. However, given the Stroop task’s widespread use as a measure of cognitive control, it is important to consider that the color-word Stroop task is testing cognitive control solely within the visual domain. This leads to the question: do these visual cognitive control findings hold for cognitive control within or across other modalities? To begin to address this question, auditory adaptations of the classic visual-word Stroop task have been implemented in previous behavioral and neuroimaging studies. One of the first studies to utilize an auditory Stroop task behaviorally required participants to indicate if they heard the word “low” or “high”, with the word spoken at either a high or low pitch [39]. The incongruent trials, in which the meaning of the word was in conflict with the pitch (e.g. the word “low” spoken in a high pitch), resulted in significant interference effects on reaction time; this auditory Stroop effect has been replicated in subsequent behavioral studies [40–42]. Roberts and Hall (2008) used a similar high/low auditory Stroop task in a functional magnetic resonance imaging (fMRI) study to directly compare the brain regions engaged during an auditory compared to a visual color-word Stroop task. This study identified common activations for interference (e.g. incongruent-neutral) in both modalities in the bilateral inferior frontal gyrus (pars triangularis), left superior parietal lobule, and the anterior cingulate cortex (ACC). However, comparing the incongruent-neutral contrast between the visual and auditory Stroop versions revealed that the visual, color-word Stroop task exhibited additional activation in the right inferior frontal gyrus (IFG) and anterior insula, while the auditory Stroop task showed a significantly larger area of activation over the left prefrontal cortex (PFC) and additional activation in the inferior parietal lobe. Christensen et al.’s [43] fMRI study also implemented an auditory Stroop test, with the task requiring gender identification of either the word or speaker instead of high/low pitch. Their paradigm elicited behavioral effects similar to previous visual and auditory Stroop studies. Interference effects (e.g. incongruent-neutral trials) were identified in the superior and inferior parietal lobules, anterior insula, anterior cingulate, and putamen, qualitatively overlapping with regions of the cingulo-opercular network often identified by visual Stroop tasks [31,34,38]. Taken together, these studies suggest that Stroop tasks recruit similar but distinct cognitive control brain networks in both the visual and auditory modalities.

Regarding cognitive control across modalities, a few behavioral studies have utilized an audiovisual (AV) color-word Stroop task to investigate crossmodal conflict. Cowan and Barron [44] utilized a paradigm in which participants performed a visual color-word Stroop task while listening to several different types of auditory distractors, including spoken words of the same color as the visual stimuli. It was found that randomly spoken color words significantly interfered with performance while other auditory distractors like non-color words and music did not. Donohue et al. [45] also investigated audiovisual conflict processing using a color-word audiovisual Stroop task. Participants were asked to identify either the printed or spoken word while being presented with the congruent, incongruent, or a neutral word in the other modality. Visual distractors of auditory targets produced interference effects, as did auditory distractors of visual targets, although the effect was largest for visual distractors of auditory targets. These two audiovisual color-word Stroop behavioral studies suggest that similar interference and facilitation effects occur crossmodally as in the larger unimodal behavioral literature. However, it remains unknown if audiovisual cognitive control in an audiovisual Stroop task is supported by the same neural resources as found in unimodal Stroop studies.

To our knowledge, no previous neuroimaging study has investigated the neural resources of cognitive control during an audiovisual color-word Stroop task. But there is a wealth of findings within the audiovisual integration and crossmodal correspondence literatures to indicate that such an investigation is warranted: concurrent auditory and visual stimuli have been found to engage additional brain regions beyond those involved in auditory or visual processing alone (for a review see [46,47]). For example, simultaneously seeing and hearing an object engages certain brain regions more than just the visual object alone (e.g. seeing a rooster and hearing a crowing noise activates AV regions more than just the image of the rooster or the sound of crowing alone) [48,49]. While there are subcortical mechanisms of multisensory integration for physical properties of stimuli (loudness, size, etc.), the brain regions identified by the audiovisual contrast described above, that are engaged by the semantic properties of multisensory integration, include posterior superior and middle temporal regions in the superior temporal sulcus (STS), more anterior regions in the superior temporal gyrus, posterior parietal cortex, and frontal areas including premotor cortex, inferior frontal gyrus, and the middle frontal gyrus [47,48,50,51]. The superior temporal regions are reliably more activated for AV congruent (e.g. a crowing rooster) than AV incongruent objects (e.g. a barking rooster) due to their role in multisensory integration, while the inferior frontal regions demonstrate more activation for AV incongruent than congruent objects as they are thought to be involved in incongruency resolution [47,52–55,55]. Notably, Laurienti et al. [56] found that the ACC was more activated by AV semantically-congruent stimuli than to AV incongruent stimuli; this is in contrast to the unimodal cognitive control literature that finds the ACC more engaged during incongruent conditions. This finding may suggest that the ACC is involved in processing simultaneous information more generally but responds differently for multimodal versus unimodal attentional control.

Another difference between the AV integration and unimodal cognitive control literatures is the involvement of superior temporal regions. AV integration reliably implicates bilateral posterior superior temporal sulcus (pSTS) regions whereas the unimodal cognitive control literature generally does not (although pSTS activation was found to be greater for audio incongruent than visual incongruent trials by Roberts and Hall [38]). It is unclear if pSTS’s involvement in the AV integration studies is due to semantic integration per se or simply the multimodal nature of the stimuli. Audiovisual speech tasks suggest that the pSTS’s involvement in multimodal tasks is not driven by semantics: several studies comparing audiovisual speech perception to auditory speech perception alone implicate the pSTS in audiovisual integration despite using non-words and syllables with relatively little semantic content [49,57–60]. Like the pSTS, early auditory cortex has been found to be more activated by audiovisual speech than auditory speech alone [49,58,61]. Altogether, the existing literature does not reach a consensus regarding the contributions of the pSTS in audiovisual cognitive control.

The findings from the AV integration and crossmodal correspondence literatures suggest that the brain regions engaged in an AV color-word Stroop task likely differ from those engaged by the traditionally-used visual Stroop task and that known AV integration regions, such as the pSTS, may be sensitive to audiovisual cognitive control demands. However, we cannot assume that an AV color-word Stroop task would engage the same regions identified to be sensitive to congruent and incongruent AV objects or speech because of the reading element of the Stroop task. Reading a word activates different visual and language regions compared to viewing an object, including the visual word form area in the inferior temporal lobe, and the angular gyrus in the inferior parietal lobe [62]. In addition, it has been shown that silent reading activates inferior frontal and parietal regions engaged in articulation as well as auditory cortex [62]. It is unclear how these reading resources and processes may be affected by concurrent congruent or incongruent auditory information.

The current study aims to characterize the brain regions engaged in an audiovisual color-word Stroop task to help answer two questions: (1) does audiovisual cognitive control recruit the same visual cognitive control regions previously identified with the widely used color-word Stroop task? And (2) are the cortical regions known to be modulated by AV integration and crossmodal correspondence sensitive to audiovisual cognitive control demands in the color-word Stroop task? Answering these questions will provide important insights into the nature of the cognitive control processes that are assessed by the widely-used color-word Stroop task, and help to bridge the AV integration, crossmodal correspondence, and cognitive control literatures (the latter of which is largely shaped by findings from visual Stroop tasks). To this end, we adapted Donohue et al.’s [45] AV color-word Stroop task for use during functional MRI. Participants were presented an auditory color word and a printed color word and asked to indicate the color word they heard via button press. Single stimulus trials (auditory only and visual only) also were included. Experiment 1 is a behavioral study of our fMRI audiovisual Stroop paradigm; the aim was to compare our accuracy and reaction times in our AV Stroop task to previous behavioral AV Stroop findings [44,45]. Experiment 2 is an fMRI study of the AV Stroop task using a sparse-sampling protocol to minimize the effects of scanner noise during the time points of interest, which is of particular concern when presenting auditory stimuli [63]. We expect that the AV Stroop task will engage both cognitive control resources identified in unimodal Stroop studies, as well as auditory regions that can be modulated by the presence of visual information. Thus, we hypothesize that:

1. AV Stroop incongruent trials will engage regions previously found in unimodal Stroop tasks to exhibit interference effects, including portions of the inferior frontal cortex, precuneus and the ACC.
2. AV Stroop incongruent trials (compared to AV congruent and unimodal trials) will engage posterior STS regions previously implicated in AV semantic integration, suggesting that these well-studied AV integration regions are sensitive to increased attentional control demands.
3. Auditory cortices (anterior to pSTS) will exhibit increased activation for AV congruent stimuli compared to auditory stimuli alone, as found in the AV speech perception literature.

## Experiment 1

### Materials and methods

#### Participants

Twenty-nine participants (all female, reflective of the student population in the corresponding author’s department) were recruited from the Department of Speech and Hearing Science at Arizona State University, Tempe campus. Participants had a mean age = 23.8 years (sd = 6.5, range = 18–48 years) and mean education = 15 years (sd = 1.3 years, range = 12–18 years). All participants self-reported to be native English speakers and absent of any neurological or psychological conditions. All participants were right-handed as determined by the Edinburgh Handedness Scale. Written informed consent was obtained from each participant prior to the study and they were compensated for their time either monetarily or by receiving course credit. The Arizona State University Institutional Review Board (IRB) approved all procedures used in this study.

#### Experimental design

Participants completed an AV Stroop task adapted from Donohue et al. [45], with four conditions: (1) *congruent*: visual presentation of a written color word (e.g. Blue) in black font and auditory presentation of the same word (e.g. “Blue”); (2) *incongruent*: presentation of a written color word (e.g. Blue) in black font accompanied by auditory presentation of a different color word (e.g. “Red”); (3) *audio-only*: only an auditory word was presented, no visual stimulus; and (4) *visual-only*: only visual presentation of the color word, no auditory stimulus.

**Fig 1:**
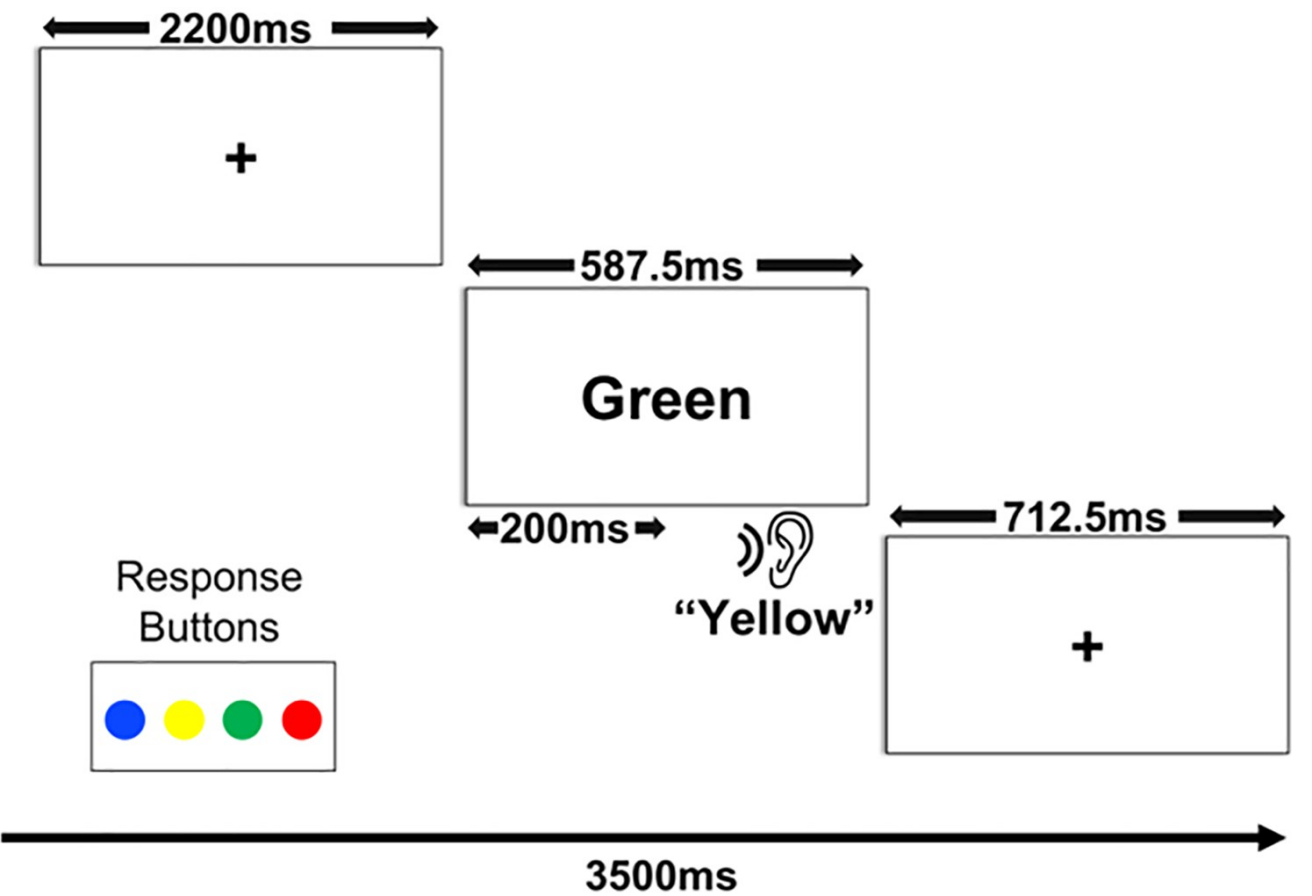
Schematic of experimental design. Visual representation of a single trial of the experimental AV Stroop task. The figure depicts an example of an incongruent trial wherein the visual and auditory words do not match.

Participants were asked to respond by pressing a button corresponding to the aurally presented word (in the case of the visual-only trials, participants were asked to respond to the written word). The words, “Red,” “Blue,” “Yellow,” and “Green” were displayed and printed in sentence case, 45-point Courier New font. Participants were instructed to respond via four adjacent keyboard buttons, each labelled to correspond to the four colors (blue, yellow, green, and red), using the four fingers of their right hand with one finger corresponding to one color’s button (see Fig 1). Prior to the start of the experiment, participants were given a brief practice session to become familiar with the response mappings in each modality individually, as well as an additional audiovisual block. In this practice session, participants were first given 12 audio-only and 12 visual-only trials during which the screen displayed a visual representation (i.e. color circles) of the button order on the keyboard to aid in response mapping. To confirm that participants correctly learned the colors associated with the keyboard buttons, 4 audio-only and 4 visual-only trials were given without the visual response aid. Participants repeated the previous 24 trials with the visual response aide and the eight confirmatory trials if needed to learn the button mapping. Next, participants practiced responding to only the auditory color stimulus in 12 congruent trials and 12 incongruent trials. Finally, participants practiced all trial types combined, with 12 incongruent trials of all AV color combinations, four congruent trials, four audio-only trials, and four visual-only trials. The practice data was not analyzed.

Each participant then completed two blocks of the experimental task. Each block had a duration of five minutes and 25 seconds. Within each block, 20 trials of each condition and 20 null trials were presented in a random order for a total of 100 trials in each block and 200 trials in total. Null trials (no stimuli presented, a fixation cross remained on the screen) were included to reduce anticipation and to replicate Experiment 2’s fMRI design. Each trial had a duration of 3.5 seconds which included: silence and a fixation cross in the middle of the screen for 2200 milliseconds, presentation of the visual and auditory words, and then silence and a fixation cross for the remaining time. Visual words in all but the audio-only condition appeared in the center of the screen for 385 milliseconds. Auditory words were spoken by a male native-English speaker and ranged in duration between 356 -414ms (*M* = 387.5ms). In the congruent and incongruent AV trials auditory words were presented 200ms after the onset of the visual word presentation. The onset of the visual word presentation preceded the auditory word by 200ms based on Donohue et al.’s [45] finding that this lag time maximized the interference effect of a visual stimulus on an auditory target.

The task was conducted on a Dell laptop in a quiet room. Auditory stimuli were presented through circumaural headphones and participants were instructed to adjust the volume to a comfortable level prior to initiating the experiment. E-Prime 2.0 software (Psychology Software Tools, Pittsburgh, PA) was used to present the stimuli and record reaction time and accuracy. Reaction times for correct trials and accuracy were each analyzed in separate one-way repeated-measures ANOVAs with four conditions (congruent, incongruent, audio-only, visual-only).

### Results

Responses that fell outside of 400 to 2500 ms post stimulus onset were excluded from both accuracy and reaction time statistics (mean = .58% of total trials excluded for each subject: .52% of incongruent, .78% of congruent, .17% of audio-only, and 0.86% of visual-only trials). The lower bound was to ensure that responses included were not premature, i.e. before participants both read the visually presented word and heard a majority of the aurally presented word. The upper bound of 2500ms was selected to ensure that the response was made prior to the beginning of the next trial.

**Fig 2:**
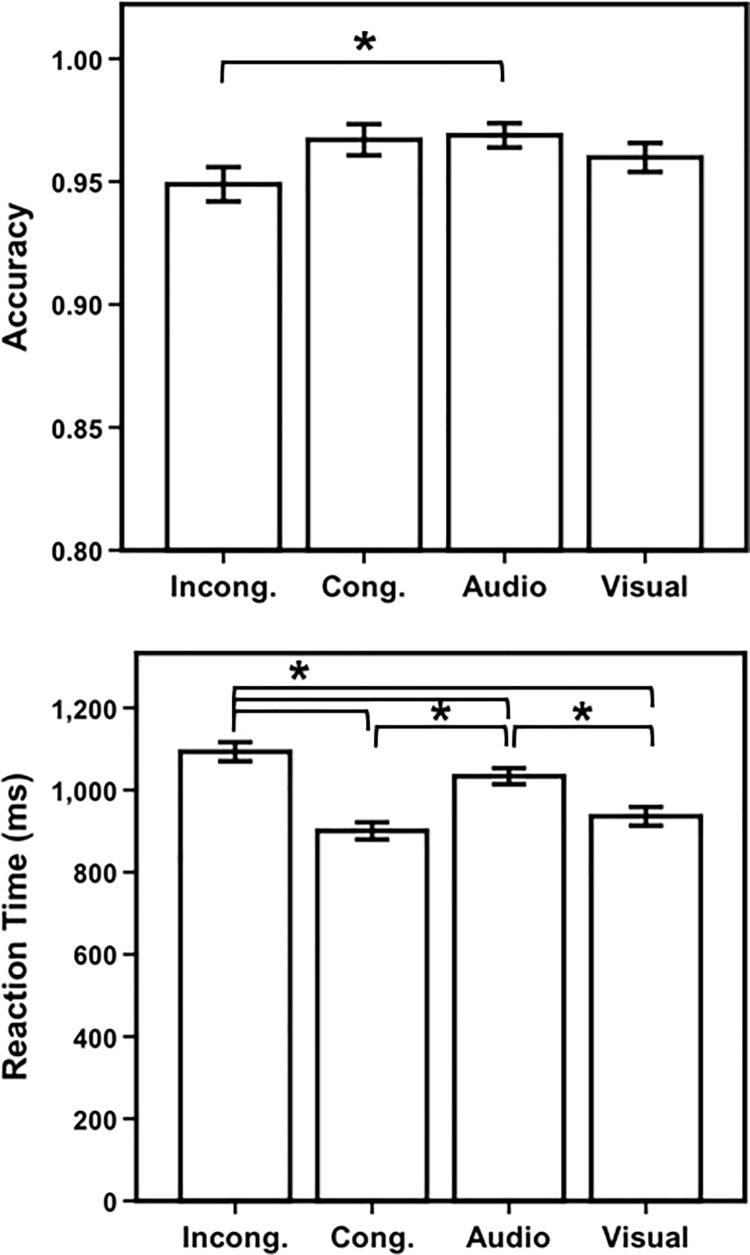
Accuracy and reaction time results from experiment 1. Average accuracy (displayed as proportion correct) and reaction time in milliseconds for each AV Stroop task condition for the behavioral participants (n = 29) in experiment 1. Conditions: Incong. = Incongruent; Cong. = Congruent; Audio = Audio-only; Visual = Visual-only. Error bars represent ± 1 standard error of the mean. *p < 0.008.

Mean accuracy results were as follows: incongruent = 94.8% (*sd* = 3.8), congruent = 96.8% (*sd* = 3.4), audio-only = 96.9% (*sd* = 2.7), and visual-only = 96.0% (*sd* = 1.8). The ANOVA for accuracy, with trial type as the factor (with four levels: congruent, incongruent, audio-only, and visual-only), revealed a significant main effect of trial type (F(3,84) = 3.4, *p* = 0.022, ƞ^2^_p_ = .11). Pairwise comparisons (Bonferroni corrected, alpha = 0.05/6 = 0.008) revealed a significant difference in accuracy between incongruent and audio trials (*t*(28) = -3.0, *p* = 0.006) (Fig 2). There was also a trend toward differences between incongruent and congruent trials (*t*(28) = 2.3, *p* = 0.029), however this did not remain significant after multiple comparison correction.

Mean reaction times (correct responses only) for each trial type were as follows: incongruent = 1093ms (*sd* = 126), congruent trials = 901ms (*sd* = 113), audio-only = 1034ms (*sd* = 105), and visual-only = 936ms (*sd* = 123) (Fig 2). Inclusion of only the correct responses in the reaction time statistics is a standard, well-studied approach in experimental psychology; the rationale is that incorrect trials should be removed to help ensure that reaction time statistics are based on trails in which normal processing has occurred [64–66]. The repeated-measures ANOVA for reaction time (factor = trial type, with four levels: incongruent, congruent, audio-only and visual-only) was significant: (F(3,84) = 89.5, *p* < 0.001, ƞ^2^_p_ = .76). Pairwise comparisons (Bonferroni corrected) of reaction times identified a significant difference between incongruent and audio-only trials (*t*(28) = 5.7, *p*< 0.001), incongruent and congruent trials (*t*(28) = 16.1, *p* < 0.001), congruent and audio-only trials (*t*(28) = -11.5, *p* < 0.001), incongruent and visual-only trials (*t*(28) = 10.5, *p* < 0.001), and visual-only and audio-only trials (*t*(28) = -5.9, *p* < 0.001). Congruent trials elicited faster reaction times than visual-only trials (*t*(28) = -2.5, *p* = .018), however this effect did not withstand multiple comparison correction.

Donohue et al. [45] used a trimming of 400–1400 ms. For purposes of comparison with Donohue et al., we report our results using this stricter trimming procedure in S1 Fig. It is noteworthy that the pairwise comparisons of reaction times across conditions that are reported to be significant remain so using either trimming procedure. Using Donohue et al.’s procedure yields two main differences: (1) it excludes substantially more trials (e.g. ~12% of incongruent trials), and (2) the accuracy main effect and incongruent to audio-only accuracy pairwise comparison reported above are no longer significant.

### Experiment 1 discussion

Experiment 1 investigated the effects of congruent and incongruent visual information on responding to an auditory stimulus in an audiovisual Stroop paradigm adapted from Donohue et al.. The overall results mostly coincide with Donohue et al.’s [45] findings. As in Donohue et al.’s [45] paradigm with a 200ms lag between visual and auditory presentation, our overall accuracy was quite high (>94% in the present study; > 92% in Donohue et al. [45]). Reaction time differences between conditions in the present study, as in Donohue et al., are greatest between incongruent and congruent conditions (*M* = 160ms and *M* = ~95ms, respectively). In the present study, congruent visual information significantly decreased reaction times compared to an auditory stimulus alone, whereas incongruent visual information significantly increased reaction times compared to an auditory stimulus alone. These facilitation and interference effects coincide with Donohue et al.’s audiovisual Stroop findings, as well as the classic “Stroop effect” found in numerous unimodal Stroop experiments [18].

The present behavioral study was not an exact replication of Donohue et al.’s [45] paradigm because we restricted our study in two ways to accommodate the MRI environment that will be necessary for Experiment 2: (i) we only included visual distractors of auditory targets as Donohue et al. found that this manipulation led to more robust interference and facilitation effects than auditory distractors of visual targets [45] and (ii) neutral AV Stroop trials (e.g. visual word distractors that were not a valid response choice) were not included. These two conditions were omitted to increase the power in the subsequent fMRI study. Due to reduced power-per-trial for sparse-acquisition fMRI compared to typical continuous-acquisition fMRI, and scanning time limitations to manage participant fatigue, including all of Donohue et al.’s [45] conditions was not feasible. Thus, we cannot discriminate between facilitation versus interference effects as often recommended in previous work (i.e. congruent versus neutral trials and incongruent versus neutral trials, respectively, [31,67]) but we did include both unimodal auditory and visual trials as did Donohue et al. to serve as baseline comparison conditions.

In summary, experiment 1 replicates previous audiovisual Stroop task findings using an abbreviated audiovisual Stroop task that is suitable for fMRI. The aim of experiment 2 was to determine the neural bases of these effects, particularly in relation to the known functional neuroanatomy of unimodal Stroop tasks and audiovisual integration.

## Experiment 2

### Materials and methods

#### Participants

24 individuals were enrolled in this fMRI experiment, however, two were excluded due to image abnormalities, one declined to complete the MRI scanning session, and one was removed from analyses due to excess motion during scanning. Thus, 20 participants (11 female) were included in the subsequent analyses: mean age = 23.9 years (*sd* = 4.7 years, range 19–37 years) and mean education = 15.9 years (*sd* = 1.7 years, range = 13–19 years). All inclusion criteria were the same as in Experiment 1 (i.e. native English speakers, right-handed, and absent of any neurological or psychological conditions), although the two participant groups did not overlap. Participants were recruited from Arizona State University, Tempe campus and the surrounding community and were monetarily compensated. Participants provided written informed consent prior to the study. The Arizona State University IRB and the St. Joseph’s Medical Center and Hospital IRB approved all procedures used in this study.

#### Experimental design

Participants completed the same AV Stroop paradigm as in experiment 1 across three fMRI scanning runs. Participants were given the same practice session as in experiment 1. In each fMRI run, participants completed 20 trials of each condition (congruent, incongruent, audio-only, visual-only, and null trials) presented in a fixed random order for a total of 100 trials per scanning run and 60 trials of each condition across the three scanning runs. Stimulus presentation and response recording were conducted using E-Prime 2.0 software (Psychology Software Tools, Pittsburgh, PA). Stimuli were delivered via Nordic Neurolab’s MR-compatible high-resolution goggles and electrostatic headphones; Nordic Neurolab’s response recording box and synchronization system was used to synchronize stimulus delivery with image acquisition.

#### fMRI data acquisition & preprocessing

Scanning was conducted at the research-dedicated 3.0 T Phillips Ingenia MRI scanner located in the Keller Center for Imaging Innovation at the Barrow Neurological Institute and St. Joseph’s Hospital and Medical Center in Phoenix, Arizona. A high-resolution anatomical T1 image was collected for each subject (MPRAGE sequence, 170 sagittal slices, FOV = 270 x 252, TR = 6.7s, TE = 3.104ms, flip angle = 90, voxel size = 1.1 x 1.1 x 1.2 mm, acquisition time = 5 minutes 34 seconds). Whole-brain functional MRI data were collected using echo-planar imaging with sparse temporal sampling parameters that follow current methodological recommendations [68]: volume acquisition time = 1804ms (leaving approximately 396ms between volume acquisition and stimulus onset), TR = 3.5s, TE = 30ms, flip angle = 90, slice thickness = 3mm, in-plane resolution = 3 x 3mm, axial slices, 103 volumes per EPI scanning run. The duration of the inter-scan interval was longer than the volume acquisition time so that auditory stimuli could be presented during moments of relative silence between the end of one volume’s acquisition and the beginning of the next volume’s acquisition. This sparse sampling technique takes advantage of the delay of the hemodynamic response to a given stimulus to reduce the relatively unknown effects of scanner noise on cognitive tasks, reduce variance in auditory cortex activation in response to auditory stimuli, and increase signal-to-noise ratio [63].

MRIcron (dmc2nii; [69]) was used to reconstruct the functional and structural images. The first three volumes from each run were removed to allow for longitudinal magnetization to reach an equilibrium. The software package Analysis of Functional Neuroimages (AFNI; http://afni.nimh.nih.gov/afni) was utilized to conduct preprocessing. Motion correction was applied to all EPI images, aligning each image to the fiftieth volume of the second run using a six-parameter rigid-body model [70]. Each participant’s anatomical image was also aligned to this volume using AFNI’s “align_epi_anat.py” program. A smoothing Gaussian kernel of 6 mm full width half maximum (FWHM) was applied to the functional images to facilitate group analyses.

#### fMRI data analysis

The software package Analysis of Functional Neuroimages (AFNI; http://afni.nimh.nih.gov/afni) was utilized to perform voxel-wise multiple regression analyses in order to investigate the brain regions engaged during each of the four experimental conditions of the AV Stroop task (AV congruent, AV incongruent, audio-only and visual-only). Regressors for the onset and duration of the auditory stimuli in each condition (or the visual stimuli, in the case of the visual-only trials) were constructed and convolved with a standard hemodynamic response function, resulting in predictor variables for analysis [71]; only trials in which a correct response was made were included in these regressors for each condition. Regressors for incorrect response trials, the grand mean of the BOLD signal, and for each of the six motion parameters also were included. An F-statistic was calculated for each voxel and statistical maps were created for each subject to identify voxels that exhibited an increased BOLD response while participants completed each condition of the AV Stroop task compared to baseline (i.e. the null trials). The statistical maps for each subject were transformed into standardized space [72] and resampled into 2 × 2 × 2mm voxels using AFNI’s “adwarp” program. The anatomical dataset for each subject was transformed into Talairach space using AFNI by manually identifying the AC-PC plane and anatomical boundaries in each subject, and then scaling each brain to the Talairach-Tourneaux brain atlas.

For the group analysis, voxel-wise *t*-tests were calculated across all subjects using AFNI’s 3dttest++ program to compare activation between conditions. Audiovisual regions have previously been classified in the literature in many ways [73]. One well-investigated technique computes the difference between the audiovisual condition and the mean of the unisensory conditions (i.e. AV > mean(A+V)), within regions that are significantly activated by both auditory and visual stimuli alone (i.e. A>0 ∩ V>0). This “mean criterion” method has been shown to effectively localize multisensory regions with less strictness than other methods (e.g. supra-additivity) [73]. Thus, we computed contrasts comparing activations during the incongruent and congruent conditions, respectively, with the mean (A+V) in A>0∩ V>0 regions. However, our goal in this study is not to just identify multisensory regions activated by our Stroop task, but to also generally investigate regions that are sensitive to multisensory cognitive control demands during our Stroop task which mostly involves making a decision about the auditory stimulus presented. These multisensory cognitive control regions may or may not be activated by a visual stimulus alone. Thus, contrasts also were computed for incongruent and congruent, respectively, versus the mean (A+V), in just A>0 regions. Contrasts of incongruent versus congruent trials, as well as each condition compared to baseline (i.e. null trials), also were computed. *T*-scores were then transformed into *z*-scores. The -clustsim option of AFNI’s 3dttest++ program was used to compute the minimum cluster-size threshold at an FWE alpha significance level of *p* < 0.05 and a voxel-wise significance level of *p* < 0.001. Minimum cluster-size threshold is reported below for each contrast.

To further explore the response properties of the regions activated more by the contrasts defined above, a region of interest (ROI) analysis was used for regions identified by the incongruent > mean (A+V), congruent > mean (A+V), and incongruent > congruent contrasts. The mean beta values within each ROI was computed for each condition compared to baseline. Within each ROI, paired t-tests comparing conditions were computed using Bonferroni multiple comparison correction (0.05/6 = 0.008).

### Results

#### Behavioral performance data

The practice data were analyzed in 19 of the 20 participants (a technical error resulted in the loss of one participant’s practice data) to ensure correct button response mapping. In the final block of the practice session (12 trials, wherein all trial types are randomly presented, mirroring the experimental procedure), participants performed with a mean accuracy of 88.8% (sd = 5.7) over all conditions indicating that the training for the button response mappings was effective. As in Experiment 1, accuracy during the experimental task was relatively high in each condition: congruent *M* = 98.1% (*sd* = 2.2), incongruent *M* = 95.8% (*sd* = 4.3), audio-only *M* = 97.4% (*sd* = 2.7), and visual-only *M* = 97.8% (*sd* = 2.5). Accuracy performances within the four AV Stroop conditions were compared using a one-way repeated measures ANOVA. The assumption of sphericity was violated using Mauchly’s test, χ^2^(5) = 15.37, *p* = 0.009, therefore Greenhouse-Geisser corrected tests are reported. Accuracy was significantly different across conditions (*F*(2.3,43.3) = 3.3, *p* = 0.04, ƞ^2^_p_ = .15). Pairwise comparisons indicate trends in the same direction as in Experiment 1; congruent and incongruent trials: *t*(19) = 2.5, *p* = 0.024, and incongruent and audio-only trials: *t*(19) = -2.2, *p* = 0.041, however these findings did not survive multiple comparison correction (Bonferroni corrected, α < .008).

#### fMRI results for each AV stroop condition

As expected, the audio-only and visual-only conditions identified both sensory regions and regions likely involved in response planning and execution: audio-only trials compared to rest (*p* < 0.001, FWE-corrected *p* < 0.05 with a minimum cluster size: 141 voxels) identified large swaths of significant activation in the bilateral superior temporal gyri (STG), as well as in the left postcentral gyrus extending into the left superior and inferior temporal lobule, and right cerebellum (Table 1). The visual-only trials compared to rest (minimum cluster size: 152 voxels) activated a large swath of voxels in bilateral occipital cortex (including bilateral lingual gyri) and left postcentral gyrus extending into the inferior parietal lobule (Table 1).

**Table 1:** Significant regions and peak coordinates for each condition compared to rest contrasts.

Region	Coordinates			Cluster Size	Peak *z*-score
	X	Y	Z		
**Audio-only > Rest**					
L Postcentral Gyrus	-49	-29	54	4234	4.1
R Superior Temporal Gyrus	63	-13	4	1182	5.1
R Cerebellum	9	-51	-2	722	3.4
**Rest > Audio-only**					
R Medial Frontal Gyrus	1	55	8	3897	4.4
L Middle Occipital Gyrus	-29	-89	8	647	3.4
R Inferior Occipital Gyrus	29	-81	-4	612	4.0
L Angular Gyrus	-47	-75	30	458	4.1
R Fusiform Gyrus	39	-61	-6	299	4.4
L Superior Frontal Gyrus	-1	23	60	223	3.4
**Visual-only > Rest**					
R Cerebellum	29	-49	-22	4413	4.7
L Postcentral Gyrus	-49	-29	54	2429	4.1
R Inferior Parietal Lobule	37	-51	44	200	4.2
R Inferior Occipital Gyrus	43	-77	-4	193	3.5
**Rest > Visual-only**					
R Medial Frontal Gyrus	1	55	6	3241	4.5
R Superior Temporal Gyrus	63	-19	8	611	4.2
R Inferior Frontal Gyrus	51	29	8	499	4.4
L Angular Gyrus	-45	-75	32	283	4.5
L Superior Frontal Gyrus	-15	41	50	245	3.8
L Superior Frontal Gyrus	1	25	60	232	3.9
L Inferior Frontal Gyrus	-51	23	4	181	3.5
**Incongruent > Rest**					
L Superior Parietal Lobule	-33	-67	54	2713	3.7
L Superior Temporal Gyrus	-65	-19	8	1121	4.4
R Cerebellum	35	-57	-22	881	3.7
L Fusiform Gyrus	-43	-55	-16	188	3.2
R Superior Temporal Gyrus	65	-23	6	499	3.5
R Precuneus	1	-71	50	262	3.4
L Lingual Gyrus	-9	-89	-2	241	3.8
R Lingual Gyrus	11	-81	0	233	3.7
**Rest > Incongruent**					
R Medial Frontal Gyrus	1	57	6	5384	5.1
L Angular Gyrus	-45	-75	32	366	3.8
R Superior Frontal Gyrus	27	27	50	186	3.9
R Middle Temporal Gyrus	43	-71	28	184	3.3
R Inferior Frontal Gyrus	29	29	-10	160	4.2
**Congruent > Rest**					
L Postcentral Gyrus	-53	-27	52	3018	4.0
R Cerebellum	19	-49	-18	1020	4.7
R Lingual Gyrus	11	-83	-2	600	3.5
R Superior Temporal Gyrus	65	-23	6	519	4.1
L Fusiform Gyrus	-39	-65	-14	338	3.5
L Lingual Gyrus	-9	-89	-2	211	3.7
**Rest > Congruent**					
L Medial Frontal Gyrus	1	57	18	2042	4.4
R Inferior Frontal Gyrus	43	17	-6	412	3.4

**Fig 3:**
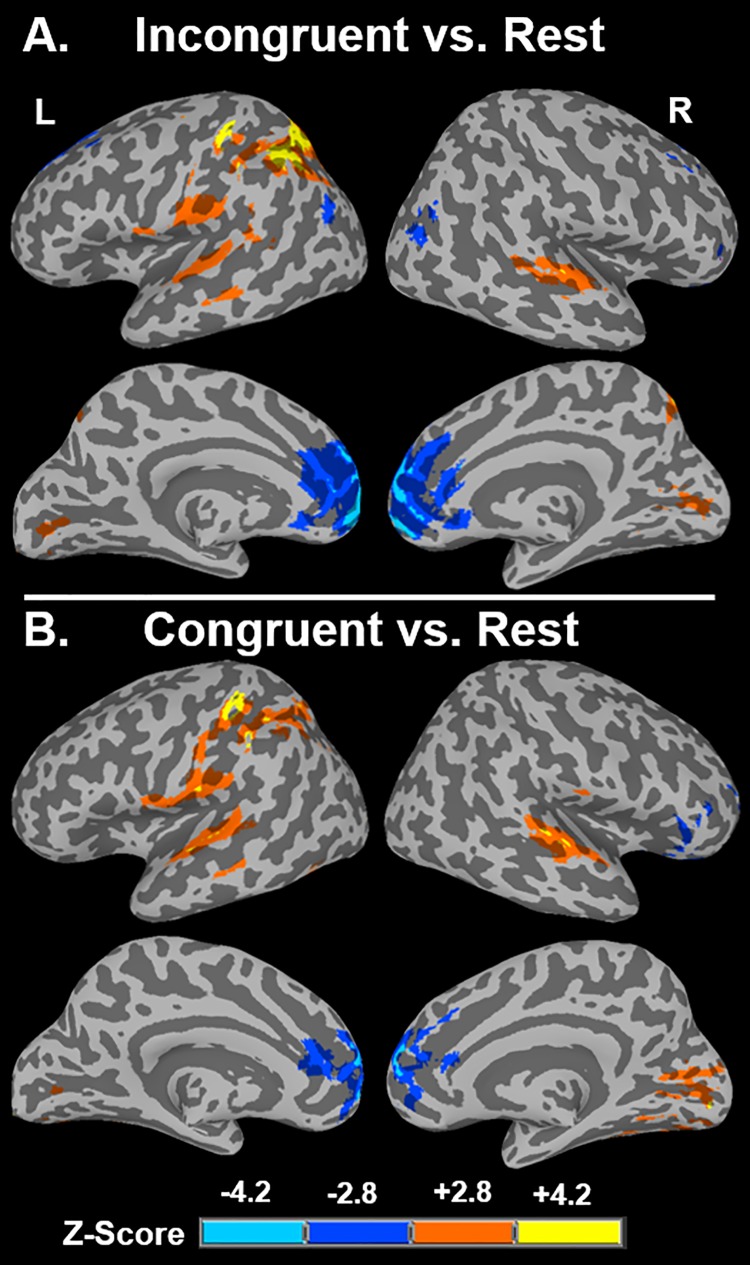
Activation for incongruent and congruent trials compared to rest. (A) Voxels whose activation is greater for AV incongruent trials than rest trials (orange and yellow) or greater for rest than incongruent trials (blue). (B) Voxels whose activation is greater for AV congruent trials than rest trials (orange and yellow) or greater for rest than congruent trials (blue). All images are displayed at voxel-wise *p* < 0.001, minimum cluster size 154 and 150 voxels, respectively, calculated by AFNI 3dClustSim.

Incongruent trials compared to rest (*p* < 0.001, FWE-corrected *p* < 0.05 with a minimum cluster size: 154 voxels) activated regions in bilateral superior and temporal and middle gyri, but notably activation in the left STG extend posteriorly. Incongruent trials also significantly activated clusters in the left superior parietal lobule extending into the left precentral gyrus and left and right precuneus. Activation was also observed in bilateral lingual gyri and right cerebellum (Table 1; Fig 3A). Congruent trials compared to rest (minimum cluster size: 150 voxels) identified increased activation in the bilateral superior and middle temporal gyri (although activation in the left STG does not extend as posteriorly as seen in the incongruent>rest contrast), left postcentral gyri extending into the left superior parietal lobule, bilateral lingual gyri, left fusiform, and right cerebellum (Table 1; Fig 3B).

#### fMRI results: AV facilitation & interference in AV integration regions

**Fig 4:**
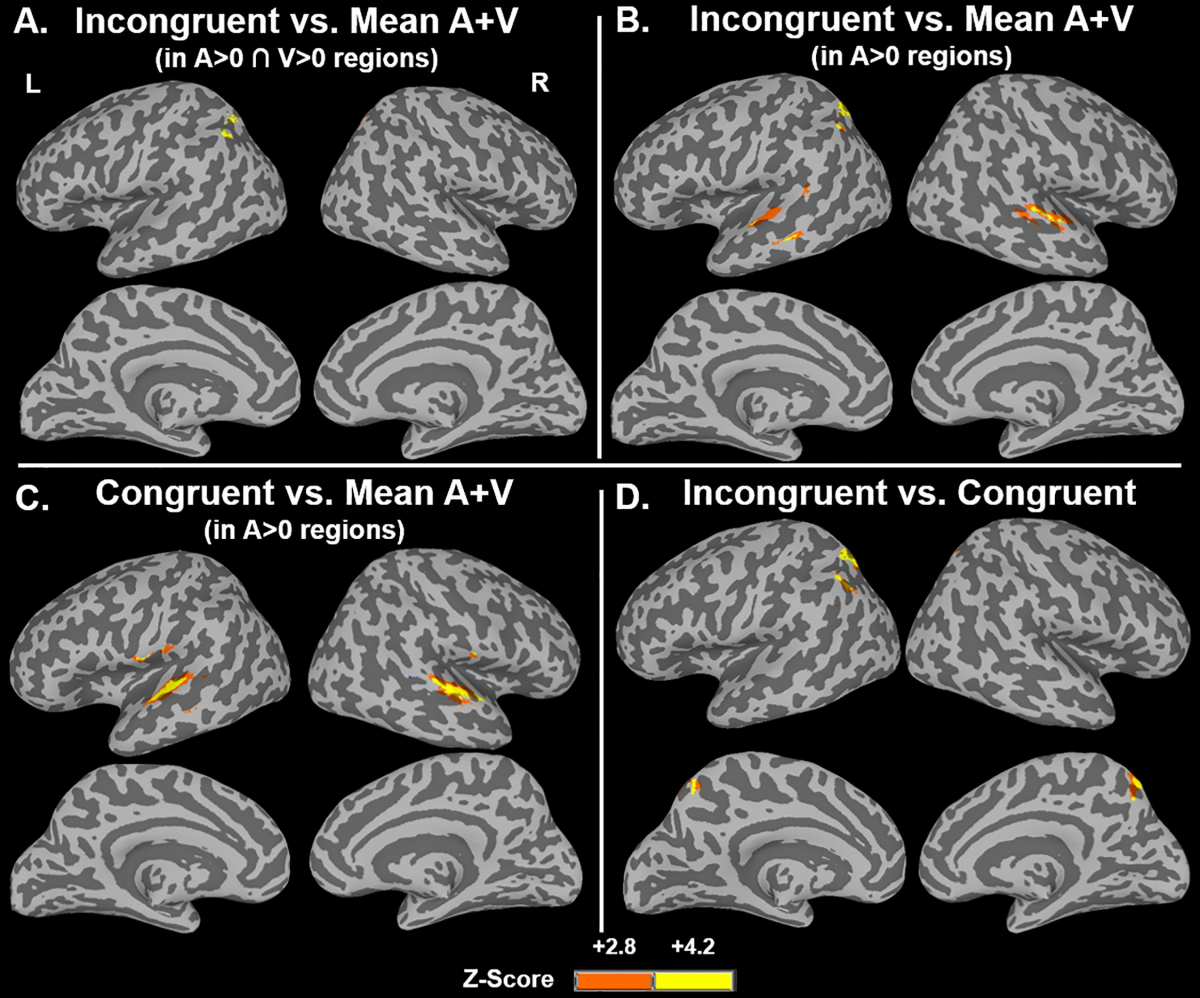
Activation for AV interference, facilitation, and conflict resolution. (A) Voxels whose activation is greater for AV incongruent trials than the mean of audio- and visual-only trials in A>0 ∩ V>0 regions. (B) Voxels whose activation is greater for AV incongruent trials than the mean of audio- and visual-only trials in A>0 regions. (C) Voxels whose activation is greater for AV congruent trials than the mean of audio- and visual-only trials in A>0 regions. (D) Voxels whose activation is greater for AV incongruent trials than AV congruent trials.

**Fig 5:**
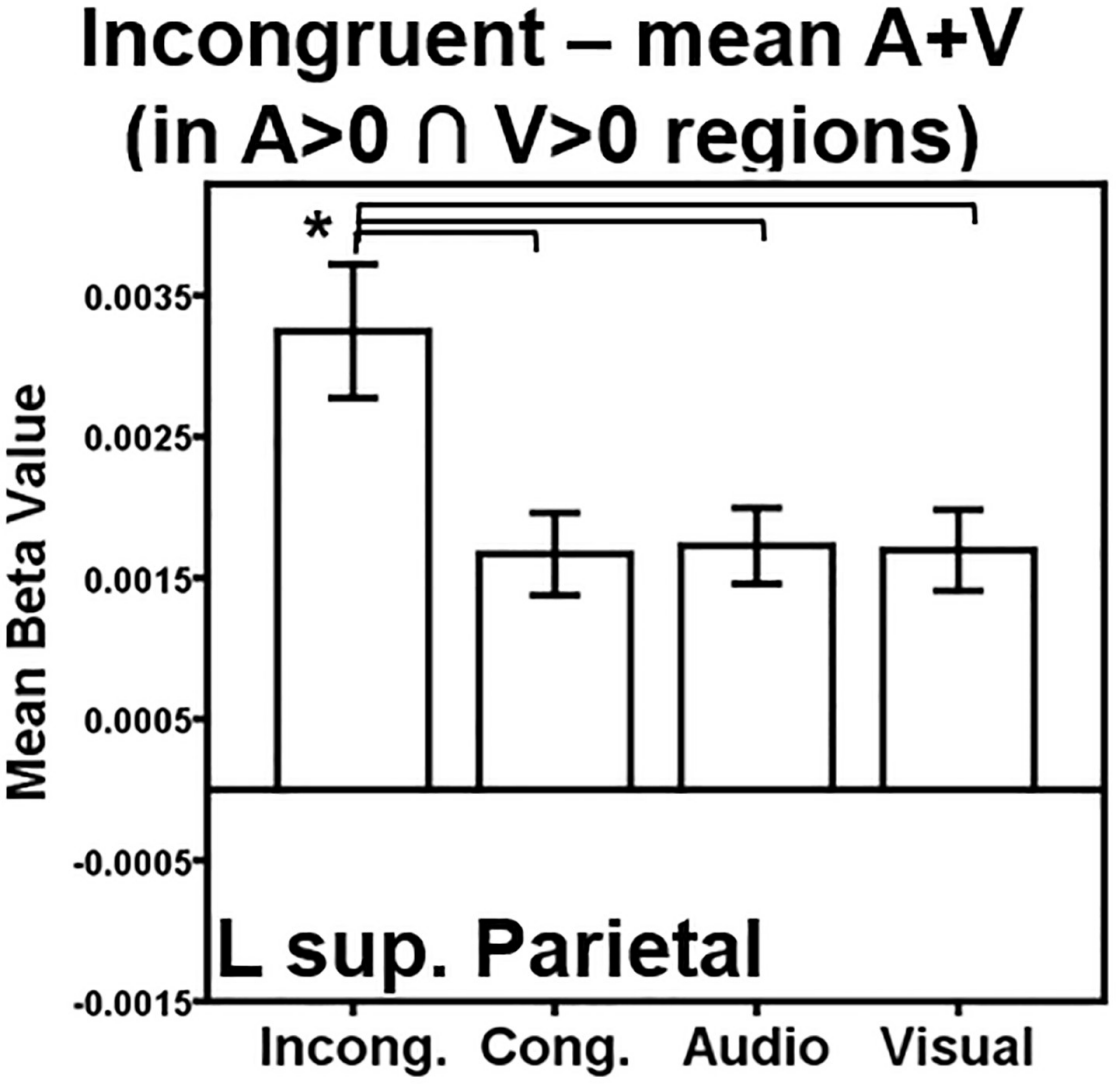
AV incongruent within A>0 ∩ V>0 ROI analysis. A region of interest (ROI) was generated from the significant region of activation in the incongruent > mean of the audio- and visual-only contrast maps within A>0 ∩ V>0 regions in Fig 4. Activation within the ROI is plotted for each condition > rest. Key: L = Left, Sup. = Superior. Conditions: Incong. = Incongruent; Cong. = Congruent; Audio = Audio-only; Visual = Visual-only. Error bars represent ± 1 standard error of the mean. *p < 0.008.

To investigate how AV cognitive control relates to multisensory regions as defined in the AV integration literature, the contrast of the AV incongruent condition versus mean (A+V) in A>0 ∩ V>0 regions was computed, as well as the contrast of the AV congruent condition versus the mean (A+V) in A>0 ∩ V>0 regions. A>0 and V>0 regions were determined by a threshold of *p* < 0.001, FWE-corrected *p* < 0.05 with a minimum cluster size of 141 and 152 voxels, respectively. The threshold for each contrast within these A>0 ∩ V>0 regions was *p* < 0.001, uncorrected. As suggested by Beauchamp [73], correction for multiple comparisons is not applied for this second criterion because it is being only being computed within the relatively small pool of voxels that passed the first criterion. The AV incongruent contrast identified one region, with its peak in the left superior parietal lobule (-31, -65, 48, 110 voxels), and extending into portions of the angular gyrus (Fig 4A). Pairwise comparisons indicate that incongruent trials elicited more activation than all other conditions (congruent *t*(19) = 4.3, *p* < 0.001; audio-only *t*(19) = 4.6, *p* < 0.001; visual-only *t*(19) = 4.4, *p* < 0.001); there were no other significant pairwise comparisons across the conditions (Fig 5). There were no regions with more activation for mean (A+V) than AV incongruent in A>0 ∩ V>0 regions. The contrast of the AV congruent condition versus mean (A+V) in A>0 ∩ V>0 regions did not identify any significant clusters.

The above contrasts are helpful in comparing our findings to the previous AV integration literature, but we also are interested in determining how auditory regions are modulated by multisensory cognitive control, and they may not be sensitive to both auditory and visual stimuli alone. Thus, we also conducted contrasts comparing the activations to the incongruent and congruent conditions to the mean (A+V), in regions significantly activated by the auditory-only condition versus rest (i.e. without the additional V>0 criterion). A>0 regions were determined by a threshold of *p* < 0.001, FWE-corrected *p* < 0.05 with a minimum cluster size of 141 voxels. The threshold for each contrast within these A>0 regions was *p* < 0.001, uncorrected. Regions significantly more active during the AV incongruent condition than the mean of audio- and visual-only conditions (presumably due to interference effects) in A>0 regions, contained peaks in the left superior parietal lobule, left posterior STG, and bilateral anterior STG (Table 2; Fig 4B). The superior parietal cluster was highly overlapping with the superior parietal clustered identified with the incongruent > mean (A+V), A>0 ∩ V>0 criteria, described in the paragraph above. There were no regions with more activation for the mean of audio- and visual-only than the AV incongruent stimuli in A>0 regions.

**Table 2:** Significant regions and peak coordinates for incongruent and congruent–mean unisensory contrasts in A>0 regions (all clusters > 10 voxels reported).

Region	Coordinates			Cluster Size	Z-Score
	X	Y	Z		
**Incongruent > mean (Audio-only + Visual-only)**					
L Superior Parietal Lobule	-31	-69	52	222	4.2
L Superior Temporal Gyrus	-65	-21	6	315	4.1
R Superior Temporal Gyrus	65	-25	6	288	3.6
**Congruent > mean (Audio-only + Visual-only)**					
L Superior Temporal Gyrus	-63	-7	6	550	4.6
R Superior Temporal Gyrus	65	-23	6	348	4.0

**Fig 6:**
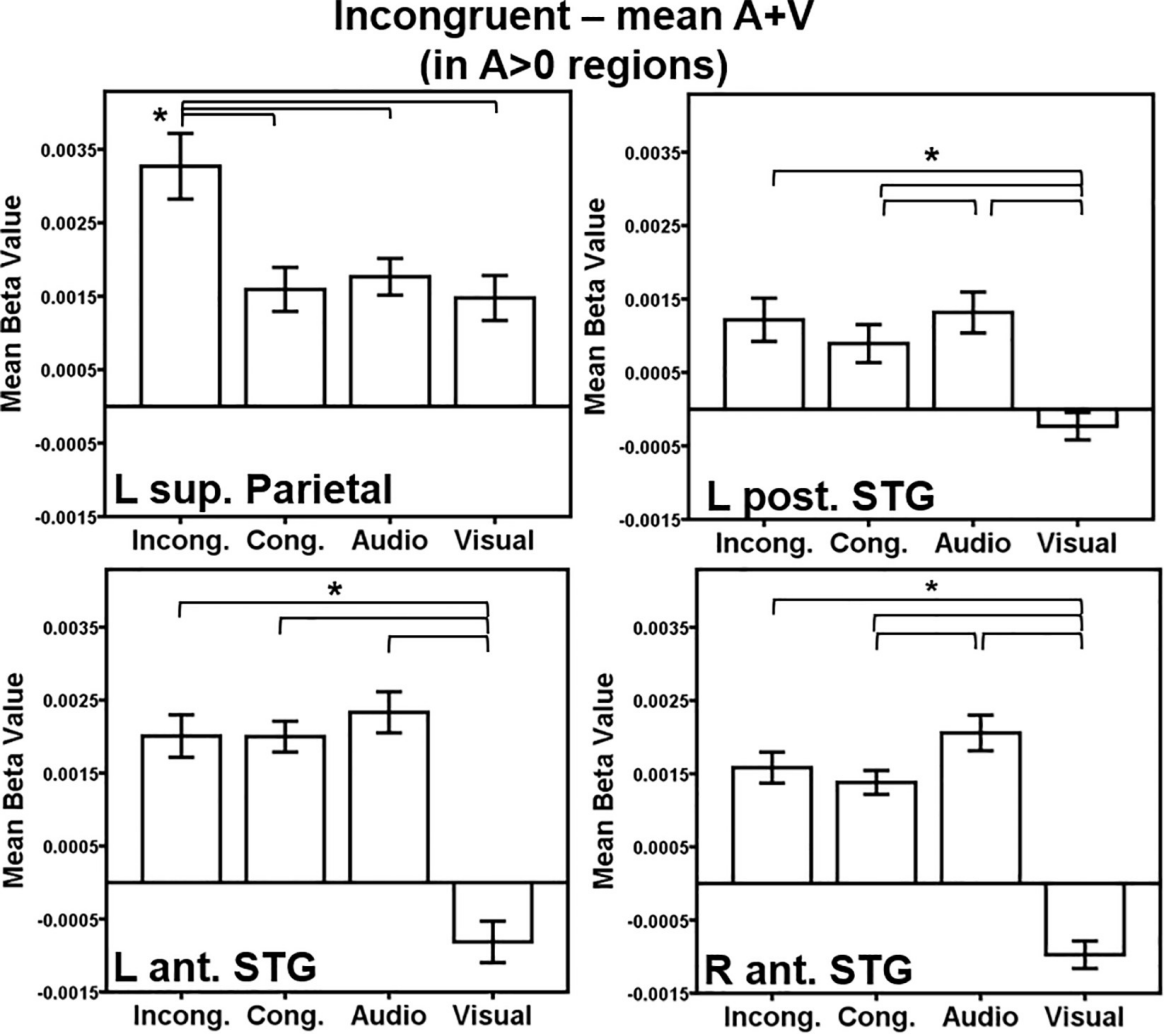
AV interference within A>0 regions ROI analysis. Regions of interest (ROIs) were generated from significant regions of activation in the incongruent > mean of the audio- and visual-only in A>0 regions in Fig 4. Activation within each ROI is plotted for each condition > rest. Key: L = Left, R = Right, Sup. = Superior, Post. = Posterior, Ant. = Anterior, STG = Superior temporal gyrus. Conditions: Incong. = Incongruent; Cong. = Congruent; Audio = Audio-only; Visual = Visual-only. Error bars represent ± 1 standard error of the mean. *p < 0.008.

Exploring the significant regions further for this AV incongruent contrast, paired t-tests revealed the following (see Fig 6): in the left superior parietal region, incongruent trials elicited more activation than all other conditions (congruent *t*(19) = 4.7, *p* < 0.001; audio-only *t*(19) = 4.6, *p* < 0.001; visual-only *t*(19) = 5.2, *p* < 0.001), suggesting that interference or conflict is driving this activation. The region in the left posterior STG demonstrated a responsiveness to auditory stimuli in general, with greater activation in the incongruent trials than the visual-only trials (*t*(19) = 5.9, *p* < 0.001), greater activation for the congruent than visual-only trials (*t*(19) = 4.1, *p* < 0.001), and audio-only greater than visual-only trials (*t*(19) = 6.1, *p* <0.001). The posterior STG region also exhibited greater activation for the audio-only trials than for the AV congruent trials, indicating a facilitation effect due to the congruent visual stimulus (*t*(19) = 3.0, *p* = 0.003). The left anterior STG ROI was more responsive to all of the conditions containing auditory stimuli compared to the visual-only condition (incongruent>visual-only *t*(19) = 9.7, *p* < 0.001; congruent>visual-only *t*(19) = 7.6, *p* < 0.001; audio-only>visual-only *t*(19) = 6.4, *p* < 0.001). The right anterior STG also demonstrated a preference for stimuli containing any type of auditory information and exhibited facilitation (i.e. reduced activation for AV congruent compared to audio-only): the right anterior STG revealed greater activation for audio-only compared to congruent trials (*t*(19) = 4.6, *p* < 0.001) and greater activation for all trial types compared to visual-only (incongruent *t*(19) = 11.7, *p* < 0.001; congruent *t*(19) = 12.4, *p* < 0.001; audio-only *t*(19) = 11.0, *p* < 0.001).

**Fig 7:**
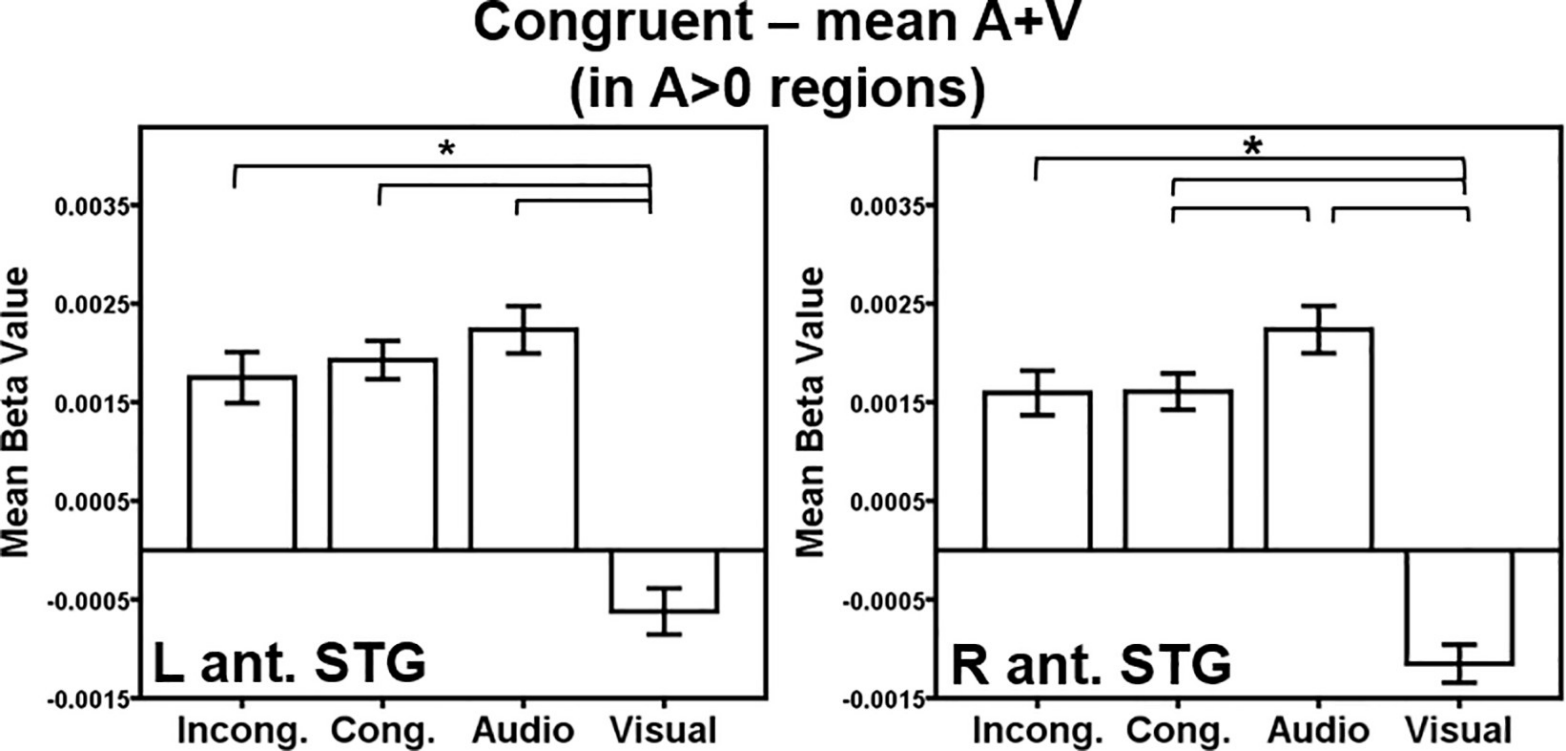
AV congruent within A>0 ROI analysis. Regions of interest (ROIs) were generated from significant regions of activation in the congruent > mean of the audio- and visual-only contrasts within A>0 regions in Fig 4. Activation within each ROI is plotted for each condition > rest. Key: L = Left, R = Right, Ant. = Anterior, STG = Superior temporal gyrus. Conditions: Incong. = Incongruent; Cong. = Congruent; Audio = Audio-only; Visual = Visual-only. Error bars represent ± 1 standard error of the mean. *p < 0.008.

A contrast of the AV congruent condition versus the mean of the audio- and visual-only condition in A>0 regions also was computed. A>0 regions were determined by a threshold of *p* < 0.001, FWE-corrected *p* < 0.05 with a minimum cluster size of 141 voxels. The threshold for each contrast within these A>0 regions was *p* < 0.001, uncorrected. Regions exhibiting greater activation for the AV congruent condition were identified bilaterally in the superior temporal gyrus, extending dorsally into the precentral and postcentral gyri (Table 2; Fig 4C). These anterior STG regions are highly overlapping with the anterior STG ROIs identified by the AV incongruent > mean (A+V) contrast in A>0 regions. ROI analyses of these anterior STG ROIs were conducted (Fig 7). In the left anterior STG, greater activation was observed for all conditions compared to visual-only trials (incongruent *t*(19) = 10.6, *p* < 0.001; congruent *t*(19) = 8.7, *p* < 0.001; audio-only *t*(19) = 7.4, *p* < 0.01); the reduced activations for AV incongruent and AV congruent trials compared to audio-only trials were not significant (*p* = 0.07 and *p* = 0.04, respectively). In the right anterior STG, all trial types also exhibited greater activation than visual-only trials (incongruent *t*(19) = 10.8, *p* < 0.001; congruent *t*(19) = 12.2, *p* < 0.001; audio-only *t*(19) = 11.1, *p* < 0.001). In addition, the audio-only trials elicited greater activation than the congruent trials (*t*(19) = 3.8, *p* < 0.001). A reduced activation to AV congruent compared to audio-only trials might suggest a facilitation effect, however, activation to AV incongruent also exhibited a similar reduced activation pattern as the AV congruent condition, although AV incongruent versus audio-only was not significant once multiple comparisons were controlled for (*p* = 0.009). This response pattern suggests that the right anterior STG’s activation to the auditory stimulus may be reduced due to the presence of any visual information, congruent or incongruent.

#### fMRI results: AV conflict resolution

We also conducted a contrast of the incongruent versus congruent conditions. While this contrast does not provide information regarding if facilitation and/or interference is driving the result, this contrast does identify regions sensitive to conflict resolution more broadly and provides a way to compare our results to those of previous unimodal color-word Stroop studies. The AV incongruent > AV congruent analysis (*p* < 0.001, FWE-corrected *p* < 0.05 with a minimum cluster size: 165 voxels) identified a significant cluster in the left precuneus and superior parietal lobule (peak = -33 63 56, peak *z* = 3.9, cluster size = 1060 voxels; Fig 4D), which highly overlaps with the superior parietal regions reported in the AV incongruent>mean audio- and visual-only contrasts reported above. There also were two regions of activation in the left posterior superior temporal sulcus (STS) including voxels in both the STG and middle temporal gyrus (peak = -55–51 14, peak *z* = 3.3, cluster size = 14 voxels) and right posterior STS (peak = 49–45 10, peak *z* = 3.6, cluster size = 79 voxels) that did not survive the multiple comparison correction for the whole brain analysis but are worthy of mention as they are located in areas of particular interest to our hypotheses and previous literature. It is important to note that the left posterior STS region identified in this contrast lies more posteriorly than the left posterior STG region identified in the AV incongruent compared to mean audio- and visual-only contrasts.

**Fig 8:**
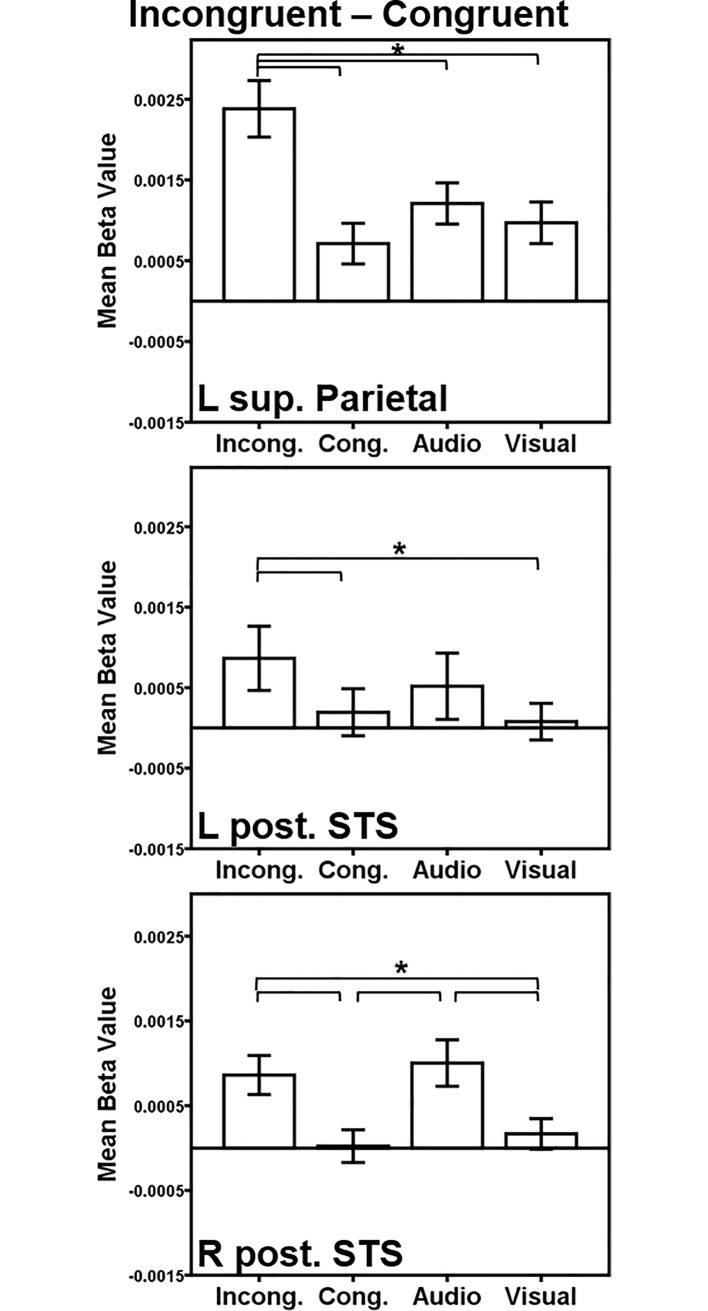
AV incongruent-congruent ROI analysis. Regions of interest (ROI) were generated from activations in the incongruent > congruent map. The posterior STS ROIs were generated from regions of activation in the incongruent > congruent map that did not survive multiple comparison correction but were of particular interest to our hypotheses. Activation within each ROI is plotted for each condition > rest. Key: L = Left, R = Right, Sup. = Superior, Post. = Posterior, STS = Superior temporal sulcus. Conditions: Incong. = Incongruent; Cong. = Congruent; Audio = Audio-only; Visual = Visual-only. Error bars represent ± 1 standard error of the mean. *p < 0.008.

Pairwise comparisons in the left superior parietal lobule ROI revealed similar findings as seen in the superior parietal ROI identified by the incongruent compared to the mean audio- and visual-only contrasts (Fig 8, top): incongruent trials elicited greater activation than all other conditions (congruent *t*(19) = 6.0, *p* < 0.001; audio-only *t*(19) = 4.8, *p* < 0.001, visual-only *t*(19) = 5.8, *p* < 0.001). While the posterior STS regions did not survive correction, an ROI analysis of these regions was still conducted due to our hypotheses in this region and the abundance of literature implicating this region in AV integration, but findings should be interpreted with caution (Fig 8, middle and bottom). In the left posterior STS ROI, incongruent trials demonstrated greater activation than congruent trials (*t*(19) = 4.0, *p* < 0.001) and visual-only trials (*t*(19) = 2.7, *p* = 0.006). In the right posterior STS, incongruent trials revealed greater activation than congruent trials (*t*(19) = 5.1, *p* < 0.001) and visual-only trials (*t*(19) = 3.6, *p* = 0.001). Further, audio-only trials showed greater activation compared to congruent trials (*t*(19) = 5.6, *p* < 0.001) and to visual-only trials (*t*(19) = 3.5, *p* = 0.001). These findings in the posterior STS suggest that facilitation (i.e. reduced activation due to AV congruency compared to an auditory stimulus alone) is driving the incongruent-congruent finding.

**Fig 9:**
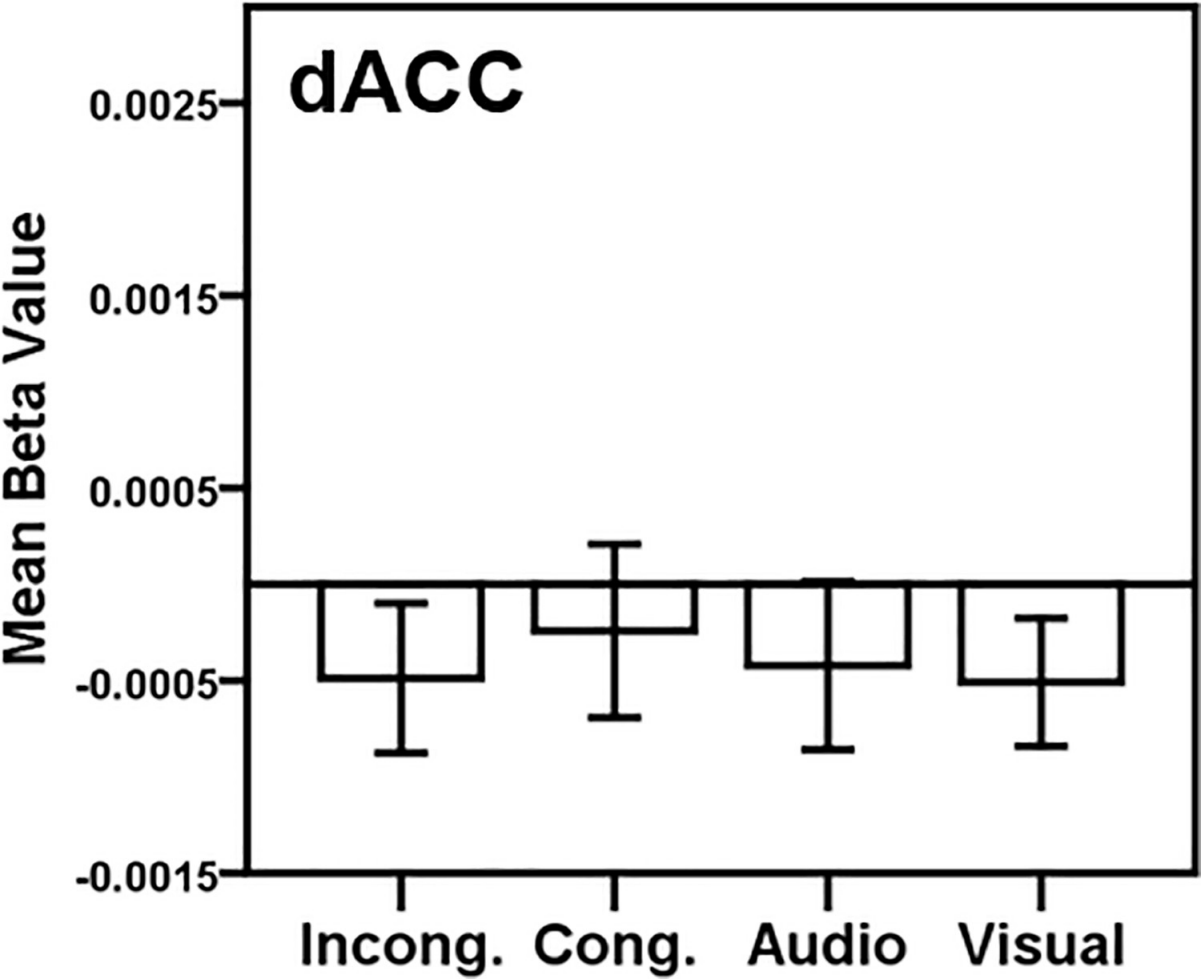
Dorsal anterior cingulate cortex ROI analysis. A region of interest (ROI) was generated from a 10 mm sphere [center Talairach coordinates 3–22 43]. Activation within the ROI is plotted for each condition > rest. Key: dACC = Dorsal anterior cingulate cortex. Conditions: Incong. = Incongruent; Cong. = Congruent; Audio = Audio-only; Visual = Visual-only. Error bars represent ± 1 standard error of the mean.

Finally, given the frequency with which dorsal anterior cingulate (dACC) activation is reported in fMRI studies of the Stroop task and our lack of significance in our data for this region, we investigated activation in the dACC in each condition (Fig 9). A spherical dACC ROI was created using AFNI’s 3dCalc program, with center of mass coordinates derived from Roberts and Hall’s [38] conjunction of fMRI findings for incongruent compared to neutral stimuli in visual Stroop and auditory Stroop tasks (Talairach coordinates: 3–22 43), and with a radius of 10mm. Paired t-tests revealed no significant differences between conditions in this dACC sphere.

## Discussion

The aim of the present study was to characterize the brain regions that support cognitive control during an audiovisual (AV) color-word Stroop task. The neurobiology of single-modality cognitive control (typically within either the visual or auditory domains) has been well studied in color-word Stroop tasks and other similar paradigms, as has audiovisual control and integration for sounds and objects. But to our knowledge, no previous neuroimaging study has investigated AV cognitive control using a color-word Stroop task. Thus, it was unclear how the brain regions recruited during the widely-used visual color-word Stroop task are dependent upon it being a unimodal task, and how color-word Stroop task results relate to AV integration and crossmodal correspondence findings. The present AV color-word Stroop fMRI study fills these gaps in the literature and provides an intersection between the fields of cognitive control and audiovisual integration. Specifically, the present study (1) characterizes how traditionally-defined AV integration regions are modulated in a classic cognitive control paradigm, and (2) identifies the neural correlates of conflict resolution in an AV color-word Stroop task, for comparison to the neural correlates of previous unimodal color-word Stroop tasks. We replicated previous behavioral AV Stroop task findings [44,45], in that congruent visual information facilitated auditory speech perception and incongruent visual information interfered with auditory speech perception (as measured by reaction time differences between congruent or incongruent trials and audio-only trials). We then implemented our fMRI-adapted version of Donohue et al.’s [45] AV Stroop task during fMRI acquisition. Our findings are discussed in detail below. Briefly, our results indicate that some AV integration regions are sensitive to AV interference while others are sensitive to facilitation in a color-word Stroop task, and that our AV color-word Stroop task engages regions distinct from those modulated by unimodal color-word Stroop tasks in previous studies.

### AV integration & cognitive control

Using a common AV integration localization procedure [73], we identified a left superior parietal region that was more sensitive to AV stimuli than unimodal stimuli. This superior parietal region exhibited an interference effect, with greater activation for AV incongruent stimuli than AV congruent or audio-only stimuli. This finding suggests that this superior parietal AV integration region is sensitive to AV attentional control demands, as it is taxed due to interference in the incongruent trials. These results coincide with those reported in fMRI studies of unimodal color-word Stroop tasks, which demonstrate parietal involvement in cognitive control processes including detection and resolution of conflicting stimuli [34,35,38,74]. The present study suggests that these findings in unimodal color word Stroop tasks may not be specific to the visual domain.

The superior parietal region described above was the only region identified by the traditional AV integration localizer, i.e. the contrast of AV stimuli versus the mean of auditory and visual-only conditions, within regions significantly activated by both auditory and visual conditions compared to rest. While investigating AV Stroop findings using the AV integration localizer is important for linking our AV color-word Stroop findings to the AV integration literature, it may not capture auditory regions that are sensitive to AV cognitive control demands. Our AV Stroop task is ultimately an auditory identification task, with visual information possibly interacting with the auditory identification process, and it would be informative to know how auditory regions are modulated by conflicting or confirming visual information. Responses of auditory areas to facilitating or interfering visual information is particularly important in order to relate the present findings to the AV speech literature: some auditory regions have been found to be more activated by AV speech than auditory speech alone [57–60], but it remains unclear if presentation of an AV congruent or incongruent written word would also modulate auditory cortex in a similar fashion. Thus, we recomputed the AV integration localizer, this time only limiting the AV versus mean (A+V) contrasts to regions significantly activated by the auditory-only condition (i.e. activation to the visual only condition was not required). These contrasts again identified the superior parietal region found to exhibit an interference effect, as described above, but also identified bilateral anterior STG regions that demonstrated AV facilitation effects: the right anterior STG region activated by auditory stimuli alone also demonstrated decreased activation for the AV congruent condition compared to the AV incongruent and auditory conditions alone. Anterior STG regions have frequently been implicated in a variety of auditory paradigms comparing normal speech to a variety of acoustically-matched controls [75–77], as well as activated by reading [78,79] and lip-reading tasks [77]. The anterior STG regions identified by our AV contrasts appear to be primarily auditory regions, as they were activated more by the audio-only condition than any other condition, and notably the visual-only condition was associated with deactivation in this region. This reduced activation in auditory cortex when visual information is present may be due to reduced attention to the auditory stimulus because of the presence of visual information, regardless of congruency. The “visual capture” effect of attention is well-documented [80,81], and it may be contributing to the reduced response properties of these anterior STG regions to the AV stimuli. However, it also is noteworthy that the congruency (or incongruency) of the visual information did not affect activation levels in these auditory areas. This finding may reflect that these anterior STG auditory regions are not mediated by top-down processes involved in conflict resolution [82–84], in contrast to posterior STG/STS auditory regions which seem to be modulated by only congruent visual information.

Posterior STG/STS areas are more commonly implicated by AV paradigms than anterior temporal regions. Multisensory stimuli have been found to bilaterally activate the posterior STS [48,57,85,86] to a greater extent than individual modalities (auditory only or visual only) [87]. In the present study, we identified regions of AV integration in a similar manner as Beauchamp et al. [73], but our AV integration localizers did not identify the pSTS regions frequently identified in the AV integration literature. However, we did find small bilateral posterior STS clusters to exhibit reduced activation in response to AV congruent versus AV incongruent stimuli. The right pSTS region identified by the AV incongruent–congruent contrast exhibited facilitation (i.e. reduced activation) in response to the AV congruent stimuli compared to the auditory-only and AV incongruent stimuli. The left pSTS region identified by the AV incongruent–congruent contrast responded in a similar fashion, but the facilitation effect was not significant. This AV incongruent–AV congruent finding was being driven by reduced activation to the AV congruent condition. These findings of facilitation in pSTS regions suggest that the presence of congruent visual information can perhaps reduce AV integration demands supported by the pSTS. This AV facilitation in pSTS would coincide with findings from the AV speech literature that incongruent AV speech stimuli (i.e. where the mouth movements seen do not match the speech sounds) elicit greater activation than congruent speech stimuli [88,89]. Together these findings suggest that AV integration resources in pSTS are not necessarily activated more when integration is difficult (e.g. incongruent AV stimuli), but rather their demand is reduced by the presence of helpful visual information.

The audio-only trials also elicited significantly more activation than visual-only trials in the pSTS region identified by the AV incongruent-congruent contrast (Fig 8), suggesting that the pSTS is primarily sensitive to auditory information. This conclusion somewhat conflicts with the AV integration literature that finds pSTS regions more activated by the combination of auditory and visual information than just auditory or visual alone [48,49,87]. It may be that this discrepancy is due to the fact that the AV Stroop task’s instructions are to attend to the auditory stimulus, whereas the AV integration studies typically attempt to engage both modalities more equally [47]. Thus, our AV Stroop task engages more of the auditory-tuned regions of the pSTS, whereas AV integration studies may engage portions of the pSTS that are more multi-sensory in nature. This would coincide with previous human and macaque work suggesting that the pSTS consists of a continuum of auditory, audiovisual, and visual subregions [85,90,91].

### AV conflict resolution

Previous unimodal Stroop studies have identified several brain regions purported to be involved in conflict resolution including the left superior parietal lobe, bilateral inferior frontal cortex, and the anterior cingulate [14,18,29,31,34–38]. As discussed above, the present AV Stroop study also implicated the bilateral superior parietal lobe. We find a left superior parietal lobe region to be active for both audio-only and visual-only conditions but is most activated by the AV incongruent condition suggesting that this superior parietal lobe is recruited by the increase in cognitive control demands. This finding combined with previous findings in the unimodal Stroop literature suggest that superior parietal cortex’s support of conflict resolution is not dependent upon a particular modality.

Our findings regarding the ACC do not align as clearly to previous unimodal cognitive control findings. We found a ventral portion of the ACC to be modulated compared to rest for both AV congruent and incongruent conditions, but the dorsal ACC region previously implicated in unimodal Stroop tasks was not modulated by any of the conditions. Thus, in the present study, the ACC was not implicated in cognitive control. Previous theories of the role of the ACC (particularly the dorsal ACC) posit that this region responds to the presence of conflict, including overriding automatic but task-irrelevant responses, as in reading the word in a color-word Stroop task [2]. But it also is the case that the ACC is a functionally diverse region and thus can be implicated in multiple aspects of anticipation, control and conflict resolution [92]. Incongruent compared to congruent or neutral conditions in unimodal color-word Stroop tasks typically show increased activation in the ACC, suggesting that this region is highly involved in controlling attention and response selection in the presence of competing information [2,14,32,93]. In addition, some studies have also shown increased activation in the ACC during congruent conditions compared to neutral conditions, suggesting instead that this region may be involved in information selection more broadly in order to maintain task goals [14]. We did not find any significant differences in the ACC between incongruent and congruent stimuli, nor in the AV incongruent versus mean auditory and visual contrasts. The AV incongruent versus rest and the AV congruent versus rest contrasts do identify ventral ACC regions as more deactivated during the tasks than rest, but it should be noted that the locations of these ACC responses are more anterior and inferior than the regions identified in the Stroop literature. It is possible that these ventral ACC responses reflect task deactivation in portions of the ACC that are part of the default-mode network, which are deactivated by the presence of almost any outward task [94,95], not the more dorsal ACC areas typically implicated in cognitive control [2,14,32,93]. While the dorsal ACC is more commonly implicated in visual Stroop tasks, Christensen et al. [43] do not find dorsal ACC activation related to cognitive control. However, Christensen et al. [43] find ventral ACC regions that exhibit interference effects in their gender-based auditory Stroop tasks, and suggest that the ventral ACC’s involvement may be related to the pragmatics and social constructs that may be related to a gender identification task. This explanation does not obviously apply to the modulations of the ventral ACC to both AV stimuli in our study. But it is clear that future work is needed to better understand the role of the ventral ACC in auditory and cognitive control paradigms, particularly because it is not frequently implicated by visual Stroop tasks.

Given the dorsal ACC’s prominence in the visual Stroop literature, we also further targeted dorsal ACC regions using an ROI approach based on the meta-analysis of Roberts & Hall: we created a 10mm radius spherical ROI centered around the peak coordinates derived from Roberts & Hall [38] conjunction of the incongruent > neutral contrast in both visual Stroop and auditory Stroop tasks [Talairach: 3–22 43]. In this more dorsal ACC ROI, there were no significant differences in activation between conditions (Fig 9). This lack of finding in dorsal ACC for incongruent versus congruent conditions conflicts with findings from the unimodal Stroop literature [14]. But it also has been proposed in the AV integration literature that the ACC’s response properties may be related to how informative a cue is, independent of conflict [96–98]. As such, our null findings in the ACC may align more with findings from AV integration studies because the task only requires attention to the auditory information; the visual information has a 50% chance of not being informative within the audiovisual trials, thus the visual cue may not be salient enough to engage the ACC. It also is possible that our AV Stroop task is not driving any robust ACC involvement because of some design differences between the present study and previous unimodal Stroop work. Additional studies with an AV (instead of unimodal) neutral condition are needed for a more direct comparison to previous unimodal Stroop studies, and future studies should also consider manipulating the ratio of incongruent to congruent trials. In this study, we had an equal number of incongruent to congruent trials per Donohue el al.’s [45] AV paradigm. However, fewer incongruent compared to congruent trials in unimodal Stroop tasks have been found to drive interference effects [2], likely caused by the sudden salience of an infrequent, conflicting visual word, which may in turn drive more ACC involvement.

Unimodal color-word Stroop tasks also frequently identify inferior frontal regions that are modulated by interference [38]. We did not find any such effects that survived the multiple comparison minimum cluster size in the IFG at the group level, although small frontal lobe activations were observed at voxel-wise *p* < 0.001 that did not survive multiple comparison correction (see S2 Fig, red and blue activations). Individual subject variability regarding the functional organization of the IFG may be contributing to this seemingly lack of IFG involvement [99]. The AV integration literature suggests that increased activity in IFG reflects increased cognitive control demands possibly related to more effortful semantic processing [47,50]. This semantic explanation of IFG involvement in AV control and integration is consistent with Hagoort’s [100] hypothesis that portions of IFG are involved in semantic unification of spoken sentences. However, it is not clear if the minimal semantic demands from single-word, AV stimuli (as seen in the present study) and sentences are utilizing the same resources within IFG [47].

Another possible contribution to the differences found between the present study’s findings and previous findings in unimodal color-word Stroop tasks and the AV integration literatures is the relative weighting of auditory and visual information within a color-word Stroop task, and at what level the two modalities are interacting. Auditory and visual signals are known to interact and be weighed differently during object construction for a variety of physical properties, including size, location, and movement [101]. Behavioral AV studies also find facilitatory effects at both perceptual and decision-making levels due to congruent auditory and visual stimuli that correspond with one another in a variety of ways, including along semantic, structural, and statistical dimensions [102]. For example, high auditory pitches correspond to high visual locations, loud sounds correspond to large objects, low voices correspond to male faces, and names spoken by forming rounded mouth movements correspond to round objects [103–106]. AV color-word Stroop tasks certainly tax semantically-mediated resources (as most reading tasks do), but also likely tax statistically-mediated correspondence, as color words and their corresponding color are often experienced simultaneously (color names on a crayon wrapper, children’s picture books, company logos, etc.). Thus, future work is needed to better understand the relative contributions of these two types of sensory interactions within color-word Stroop tasks.

## Conclusion

In the present study we used fMRI to investigate the neural resources engaged during an audiovisual color-word Stroop task. Superior parietal regions frequently implicated in unimodal Stroop tasks and attention more generally were more responsive to AV stimuli than unimodal stimuli and exhibited an interference effect when incongruent visual information was presented alongside the auditory stimulus. Posterior STG/STS regions previously implicated in AV integration and more anterior STG auditory regions also were found to be more responsive to AV than auditory stimuli alone, but unlike the superior parietal region, these temporal regions exhibited facilitation effects (i.e. reduced activation for AV congruent stimuli than unimodal stimuli), suggesting that helpful visual information was reducing the demands of these primarily auditory regions. The dorsal ACC, which is reliably implicated in conflict resolution by unimodal Stroop tasks, was not found to be modulated by task condition in the present AV Stroop study. This null finding in the dorsal ACC may be driven by the equal distribution of congruent and incongruent trials which has been found to diminish interference effects in the ACC, but future studies are needed to clarify the role of the ACC in multimodal conflict processing. Altogether, the present study indicates that regions implicated in audiovisual integration also are sensitive to conflict resolution in a color-word Stroop task; and that an AV color-word Stroop task implicates distinct but overlapping cognitive control resources identified by previous unimodal Stroop studies. These findings suggest that AV cognitive control and AV integration rely upon overlapping resources (particularly in the superior parietal lobe). Furthermore, the visual color-word Stroop task that is widely-used as both a clinical and research measure of cognitive control, may not be capturing all of the neural resources engaged in the conflict resolution that occurs in everyday life, due to the tight connection between auditory and visual information in the real world [101].

## Supporting information

S1 Fig Accuracy and reaction time results from experiment 1 using donohue trimming procedure. Donohue et al. [45] used a trimming of 400–1400 ms. For purposes of comparison with Donohue et al., we also report our results using this stricter trimming procedure. Average accuracy (displayed as proportion correct) and reaction time in milliseconds for each AV Stroop task condition for the behavioral participants (n = 29) in experiment 1 are shown. Conditions: Incong. = Incongruent; Cong. = Congruent; Audio = Audio-only; Visual = Visual-only. Error bars represent ± 1 standard error of the mean. *p < 0.008.
(TIF)

S2 Fig Activation conjunction map. Conjunction map for voxels whose activation is: greater for AV incongruent trials than the mean of audio- and visual-only trials (red); greater for AV congruent trials than the mean audio- and visual-only trials (yellow); greater for AV incongruent trials than AV congruent trials (blue). Overlap is shown for AV incongruent trials greater than the mean of the audio- and visual-only trials overlapping with AV congruent trials greater than the mean of audio- and visual-only trials (orange) and for AV incongruent trials greater than the mean of audio- and visual-only trials overlapping with AV incongruent trials greater than AV congruent trials (purple). All images are displayed at uncorrected voxel-wise p < 0.001.
(TIF)

S1 File Fitzhugh_AVstroop_SupportingInfo.zip. Group contrast fMRI data in nifti format, region of interest values for each condition, and behavioral results (accuracy and reaction times).
(ZIP)
