# Supplementary figures and images for: A functional MRI investigation of crossmodal interference in an audiovisual Stroop task
Source: PLoS One. 2019 Jan 15;14(1):e0210736. doi: 10.1371/journal.pone.0210736 (PMC6333399; doi:10.1371/journal.pone.0210736)

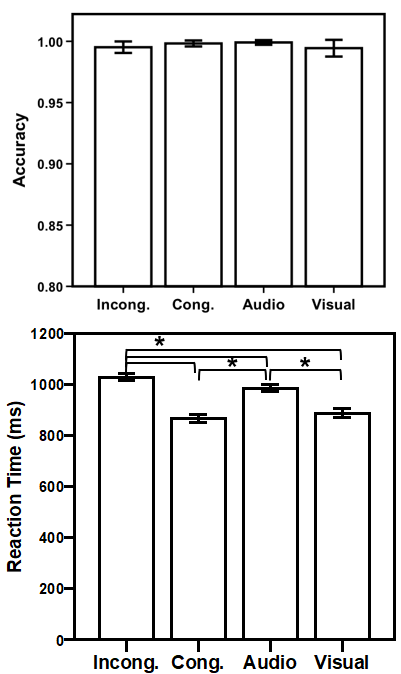

Supplement: S1 Fig — Donohue et al. [45] used a trimming of 400–1400 ms. For purposes of comparison with Donohue et al., we also report our results using this stricter trimming procedure. Average accuracy (displayed as proportion correct) and reaction time in milliseconds for each AV Stroop task condition for the behavioral participants (n = 29) in experiment 1 are shown. Conditions: Incong. = Incongruent; Cong. = Congruent; Audio = Audio-only; Visual = Visual-only. Error bars represent ± 1 standard error of the mean. *p < 0.008. (TIF) [file pone.0210736.s001.tif]

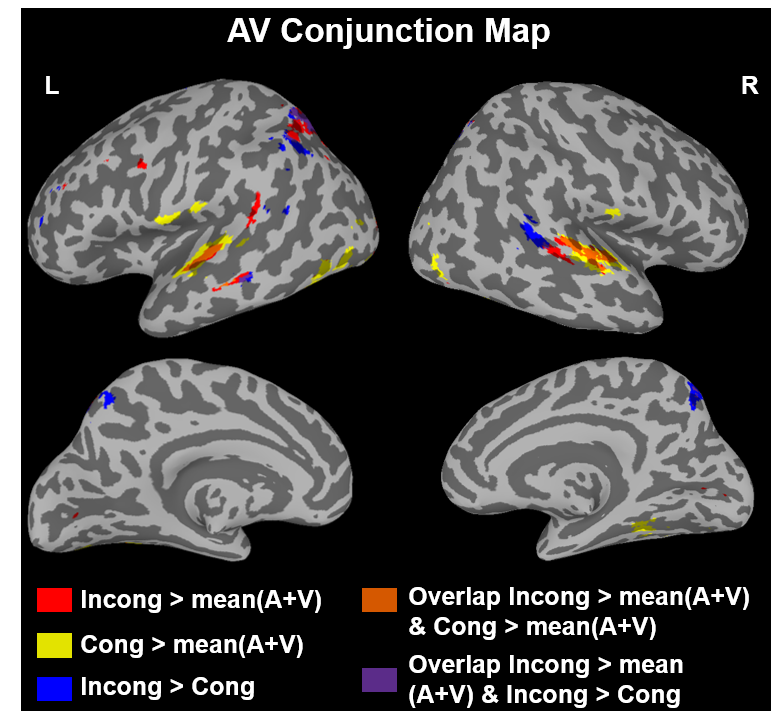

Supplement: S2 Fig — Conjunction map for voxels whose activation is: greater for AV incongruent trials than the mean of audio- and visual-only trials (red); greater for AV congruent trials than the mean audio- and visual-only trials (yellow); greater for AV incongruent trials than AV congruent trials (blue). Overlap is shown for AV incongruent trials greater than the mean of the audio- and visual-only trials overlapping with AV congruent trials greater than the mean of audio- and visual-only trials (orange) and for AV incongruent trials greater than the mean of audio- and visual-only trials overlapping with AV incongruent trials greater than AV congruent trials (purple). All images are displayed at uncorrected voxel-wise p < 0.001. (TIF) [file pone.0210736.s002.tif]
